# Supplementary material for: FastqPuri: high-performance preprocessing of RNA-seq data
Source: BMC Bioinformatics. 2019 May 3;20:226. doi: 10.1186/s12859-019-2799-0 (PMC6500068; doi:10.1186/s12859-019-2799-0)
Supplement: Supplementary file 2 — Archive of FastqPuri. Archive containing all files needed to install and run FastqPuri v1.0.6. Date stamp March 22, 2019. (GZ 47,819 kb) [file 12859_2019_2799_MOESM2_ESM.gz › FastqPuri-1.0.6/html/trimDS_8h.html]

FastqPuri: include/trimDS.h File Reference


FastqPuri


- include

Classes |
Typedefs |
Functions

trimDS.h File Reference

trim adapters from double stranded data
More...

`#include "fq_read.h"`  
`#include "defines.h"`

Include dependency graph for trimDS.h:

This graph shows which files directly or indirectly include this file:

Go to the source code of this file.

|  |  |
| --- | --- |
| Classes | |
| struct | \_ds\_adap |
|  | structure containing an adapter pair (for read 1 and read 2) More... |
|  | |

|  |  |
| --- | --- |
| Typedefs | |
| typedef struct \_ds\_adap | DS\_adap |
|  | structure containing an adapter pair (for read 1 and read 2) |
|  | |

|  |  |
| --- | --- |
| Functions | |
| DS\_adap | init\_DSadap (char \*ad1, char \*ad2, int L1, int L2) |
|  | initialization of a DS\_adap structure More... |
|  | |
| int | trim\_adapterDS (DS\_adap \*ptr\_DSad, Fq\_read \*r1, Fq\_read \*r2) |
|  | trim the sequences, discard them or keep them unchanged depending on them having adapters remnants. More... |
|  | |

## Detailed Description

trim adapters from double stranded data

Author
:   Paula Perez paula.nosp@m.pere.nosp@m.zrubi.nosp@m.o@gm.nosp@m.ail.c.nosp@m.om

Date
:   05.10.2017

## Function Documentation

## ◆ init\_DSadap()

|  |  |  |  |
| --- | --- | --- | --- |
| DS\_adap init\_DSadap | ( | char \* | *ad1*, |
|  |  | char \* | *ad2*, |
|  |  | int | *L1*, |
|  |  | int | *L2* |
|  | ) |  |  |

initialization of a DS\_adap structure

Parameters
:   |  |  |
    | --- | --- |
    | ad1 | adapter 1 sequence |
    | ad2 | adapter 2 sequence |
    | L1 | adapter 1 sequence length |
    | L2 | adapter 2 sequence length |

Returns
:   initialized DS\_adap structure

## ◆ trim\_adapterDS()

|  |  |  |  |
| --- | --- | --- | --- |
| int trim\_adapterDS | ( | DS\_adap \* | *ptr\_DSad*, |
|  |  | Fq\_read \* | *r1*, |
|  |  | Fq\_read \* | *r2* |
|  | ) |  |  |

trim the sequences, discard them or keep them unchanged depending on them having adapters remnants.

Returns
:   0 if the read is to be discarded, 1 if left as is, 2 if trimmed. Reads are trimmed if 2 is returned.


---

Generated on Mon Mar 19 2018 23:42:01 for FastqPuri by  

 1.8.14
